# IN THE PALM OF OUR HAND: HUMAN-LED ADMINISTRATIVE DATA LINKAGE TO AUGMENT LONGITUDINAL AGING STUDIES

**DOI:** 10.1093/geroni/igae098.2073

**Published:** 2024-12-31

**Authors:** Ashley N Dorame, Mina Antic, Joseph Ferrie, Robert Waldinger, Avron Spiro III, Daniel Mroczek, Lewina Lee

**Affiliations:** Boston University School Of Medicine, Boston, Massachusetts, United States; Massachusetts General Hospital/Harvard Medical School, Boston, Massachusetts, United States; Northwestern University, Evanston, Illinois, United States; Harvard University, Cambridge, Massachusetts, United States; VA Boston Healthcare System, Boston, Massachusetts, United States; Northwestern University, Evanston, Illinois, United States; Boston University Chobanian & Avedisian School Of Medicine, Boston, Massachusetts, United States

## Abstract

A robust literature has documented associations of adverse early experiences with poor lifespan health (Hughes et al., 2017). The evidence with respect to aging-related outcomes has been largely derived from population-based longitudinal studies, which predominantly rely on retrospective assessments of childhood experiences. Given the modest agreement between prospective and retrospective measures of early life exposures (Baldwin et al., 2019), it is unknown whether the well-established associations of early adversity with later-life health may replicate in prospective designs. This presentation uses the Boston Early Adversity and Mortality Study (BEAMS) to illustrate a novel approach leveraging recently-digitalized, full-count U.S. Censuses from 1900 to 1940 to obtain prospective data on the early-life family and neighborhood conditions for participants in three, all-male longitudinal aging studies (Normative Aging Study, 1961+, n=2280; Harvard Study of Adult Development, 1938+: Grant cohort, n=458, Glueck cohort, n=267). The authors will describe key steps of the human-led data linkage (“hand-linkage”) process, and present results on linkage performance and value added to the longitudinal studies. Hand-linkage identified 10,146 female and male siblings of the original cohort members. Among original cohort members born prior to each Census, hand-linkage identified 68.6% (1900), 76.3% (1910), 88.5% (1920), 88.0% (1930), and 87.9% (1940) participants, compared to match rates of 24%-36% via a commonly used algorithm (Abramitzky et al., 2021 with Jaro-Winkler adjustment). Relative to hand-linkage, the sensitivity and specificity of the algorithmic approach was 31.2% and 98.2%, respectively. Findings support human-led linkage as an effective strategy to augment the scientific value of longitudinal aging studies.

